# Harnessing Artificial Intelligence to Address Oral Health Disparities

**DOI:** 10.1001/jamahealthforum.2024.0642

**Published:** 2024-04-05

**Authors:** Hawazin W. Elani, William V. Giannobile

**Affiliations:** Department of Oral Health Policy and Epidemiology, Harvard School of Dental Medicine, Boston, Massachusetts; Department of Health Policy and Management, Harvard T. H. Chan School of Public Health, Boston, Massachusetts; Department of Oral Medicine, Infection, and Immunity, Harvard School of Dental Medicine, Boston, Massachusetts

Oral health care has been plagued by health disparities for decades. Despite multiple calls to action, the stubborn persistence of socioeconomic, racial, and ethnic disparities in oral health makes dental health among the most underserved areas of medicine.^1^

Artificial intelligence (AI) and machine learning (ML) are at the forefront of nearly every facet of health care. As with the rest of medicine, AI is permeating dentistry to improve dental care and patient outcomes. In November 2023, more than 400 AI experts, dentists, and technology developers convened at Harvard University for a global symposium on the potential of AI to transform oral health care.^2^ The symposium underscored how the future of AI in dental care will depend on collaboration among clinicians, research institutions, industry partners, and regulatory bodies.

## Can AI Help Achieve Equity in Oral Health?

Dentistry has an opportunity to embrace AI and chart the course for other areas of medicine in reducing health disparities for 3 pragmatic reasons. First, while dentistry and medicine use AI to understand diseases at a molecular level by analyzing patient biological specimens, including imaging, electronic health records, and genetic and microbiome data, dentistry has unique attributes that could make it a key focus of AI development. For example, biomedical imaging is used for routine patient care, with nearly 100 million dental radiographs taken annually in the US.^3^ Salivary biomarker evaluation is a noninvasive tool enabling early diagnoses of diseases and monitoring therapeutic response. Integrating radiographic and biomarker information with clinical and socioeconomic data makes dentistry a fertile ground to apply advanced analytics, such as multimodal AI, to enable more precise diagnoses, targeted interventions, and ultimately, improved population oral health.

Second, dentistry is a discipline that primarily focuses on reconstructive care. Dentists are trained and incentivized by a fee-for-service payment model to repair dental disease rather than prevent it. As a result, financial barriers to dental care are the highest compared with any other form of health care. However, AI-powered technologies can identify high-risk populations to prevent the initiation and progression of dental disease, such as periodontal disease and subsequent tooth loss, and assist in behavior change, leading to a shift toward prevention and minimally invasive care. ML is also used for designing biopolymers and peptides to manage microbiomes and develop vaccines to target oral pathogens. These advancements could improve care and lessen the need for costly dental procedures. However, equitable access must be ensured, especially for underrepresented populations.

Third and above all, inequitable access to dental care has been a long-standing challenge, particularly for low-income, uninsured, and racial and ethnic minority populations. These groups experience a higher burden of dental disease and face more barriers to accessing dental services.^1^ AI-powered solutions promise to expand access to dental care in several ways. By automating administrative tasks—eg, scheduling, billing, and insurance processes, including licensing and preauthorization—AI can save health care institutions billions of dollars^4^ and overcome the administrative encumbrance associated with participating in public insurance programs, encouraging more dentists to treat underserved populations. Currently, more than 75 million US residents live in dental professional shortage areas.^5^ In these areas, AI can empower mid-level clinicians, such as dental therapists, by analyzing imaging and clinical data to support decision-making.^6^ Teledentistry has been reducing the need for frequent in-person dental visits and allows for patient triaging and prescription medications, enabling populations in underserved communities to access dental services.^7^ Lastly, the growing availability of large language models is a promising opportunity to address disparities in oral health literacy. AI can disseminate tailored information to promote the importance of oral health, improve adherence to appointments, and assist in navigating the complex dental insurance structure.

## Challenges for Achieving Oral Health Equity With AI

Since 2020, the US Food and Drug Administration (FDA) has approved 478 AI and ML-enabled medical devices.^8^ However, with the rapid development of AI tools, limited explainability of AI models, and limited regulatory oversight beyond the FDA, concerns regarding bias and data privacy persist. In addition, dentists have been slow to adopt AI technologies. In a 2023 survey of 250 experienced dentists, 35% reported adopting AI tools.^9^ Most importantly, in dental care, where socioeconomic, racial, ethnic, and geographic barriers are highly pronounced, there is a higher risk of perpetuating existing health disparities through algorithmic bias in the development of AI tools and unequal access to AI-enabled technologies. As regulatory guidance on best practices for AI evolves, it will be imperative to ensure that equity is at the forefront of every AI tool development, deployment, and monitoring to avoid reinforcing disparities and mirroring the structural racism present in the existing dental health care systems.

## Stakeholders for AI in Dental Medicine

Industry developers are the key players advancing AI to assist dental practices in diagnosis, personalized therapy, and remote monitoring. Although academic institutions and payers are also involved, their contribution is limited. For AI to reshape dental care, all these parties must ensure the responsible adoption of AI-driven tools (Table).

Academic dental institutions have a responsibility to evaluate the safety and efficacy of competing AI technologies. However, this effort is hindered by limited infrastructure resources, a shortage of AI-trained personnel, and a lack of integration between dental and medical information in electronic health records. Schools of dentistry need to embrace innovative academic-industry partnerships with AI tools to engage patients, practitioners, and third-party payers. Dental education curricula are integrating AI to educate future dentists on its responsible use in daily clinical practice.^10^ It will be equally important to engage practicing dentists, hygienists, and dental faculty through continuing education and collaboration with industry AI developers.

As AI is further integrated into health care, dental medicine can leverage AI to advance oral health. However, dental professionals must first commit to sharing the responsibility of safeguarding the use of AI to improve oral health for all.

## Figures and Tables

**Table.** Opportunities for Dentistry to Use Artificial Intelligence (AI) to Advance Oral Health Care Delivery and Reduce Disparities

Stakeholder	Opportunity
Dental clinicians	Diagnostics assistance, more personalized dentalcare, targeted interventions AI-assisted and robotic-driven reconstructive technologies Remote monitoring and teledentistry
Patients and families	Remote monitoring through wearable and handheld imaging devices Engagement with professionally trained virtual assistants and teledentistry
Public and private insurers	Improve administrative efficiencies including claims processing, preauthorization, and reimbursements Premiums adjustment for indemnity insurers
Oral health care clinics and delivery systems	Optimize scheduling and billing processes Implement clinical decision support tools and teledentistry
Academic institutions	Offer AI training programs and continuing education courses Invest in data management and computing infrastructure Innovate in industry partnership
Funders	Support AI-focused funding mechanisms for capacity-building and scalability Encourage innovation and collaboration across dentistry, medicine, and industry partners
Government agencies	Develop guidelines for ethical, transparent, and fair AI use in oral health Ensure compliance with regulatory measures
